# A comparative study of antenatal depression among urban and rural pregnant women in Gimbi District, Oromia, Ethiopia

**DOI:** 10.3389/fpubh.2024.1393880

**Published:** 2024-11-08

**Authors:** Solomon Chala, Markos Desalegn, Rut Oljira, Meseret Belete Fite, Sagni Hambisa Mecha, Gemechis Megnaka Hunde

**Affiliations:** ^1^West Wollega Health Office, Gimbi, Ethiopia; ^2^Department of Public Health, Institute of Health Sciences, Wollega University, Nekemte, Ethiopia; ^3^Department of Pharmacy, Institute of Health Sciences, Wollega University, Nekemte, Ethiopia

**Keywords:** antenatal, perinatal, depression, pregnant, women

## Abstract

**Background:**

Antenatal depression (AND) is a form of clinical depression that can be caused by stress and worries that can bring pregnancy to more severe levels. It has negative impacts on women, the family, and the community at large. The comparative study of antenatal depression among rural and urban pregnant women was less studied in Ethiopia and in this study area in particular.

**Objective:**

The objective of the study was to compare the prevalence of antenatal depression and its associated factors among pregnant women in Gimbi rural and urban residents in Ethiopia in 2023.

**Methods:**

A community-based comparative cross-sectional study design was used from 1 February to 30 March 2023. A systematic random sampling method was used to select study participants. Data were collected using pretested interviewer-administered structured questionnaires. Binary logistic regression analysis was used to identify factors associated with antenatal depression. Variables with a *p*-value of 0.25 or less in the bi-variable logistic regression model were candidates for a multi-variable logistic regression model.

**Results:**

The prevalence of antenatal depression was 56 (21.5%), 95% CI: [16.9–25.5] among rural participants and 50 (19.2%) [95%] CI: [14.6–23.8] among urban participants. Having complications during pregnancy (AOR: 4.92, 95% CI: 1.35, 17.88), ever had depression (AOR: 3.20, 95% CI: 1.30, 7.85), consuming alcohol (AOR: 3.78, 95% CI: 1.24, 11.49), and educational status (can read and write) (AOR: 2.14, 95% CI:1.05, 4.67) were factors associated with antenatal depression among urban mothers, while no antenatal care follow-up (AOR: 6.6, 95% CI: 2.63, 16.85), unplanned pregnancy (AOR: 4.51, 95% CI:1.10, 1.86), and having complications during pregnancy (AOR: 2.77, 95% CI: 1.30, 5.92) were factors associated with antenatal depression among rural mothers.

**Conclusion:**

The prevalence of antenatal depression among rural mothers was higher than the prevalence of antenatal depression among urban mothers in the Gimbi district. Having complications during pregnancy, ever had depression, consuming alcohol, and educational status were associated factors with antenatal depression among urban mothers; having complications during pregnancy, unplanned pregnancy, and no ANC follow-up were factors associated with antenatal depression among rural mothers. Therefore, quality family planning and ANC services should be provided for the women to reduce unplanned pregnancies and experience complication-free pregnancy periods.

## 1 Background

Depressive disorders are defined as sadness, loss of interest or pleasure, feelings of guilt or low self-worth, disturbed sleep or appetite, feelings of tiredness, and poor concentration (1). The most prevalent mental disorder during pregnancy is antenatal depression (2). Antenatal depression is a form of clinical depression caused by stress and can cause worry during pregnancy (3). Depression during pregnancy is a prevalent mental health problem affecting approximately one in five women worldwide (4). It is considered to be a serious public health concern in the developing world that hinders maternal health improvement and is predicted to become the most common cause of disability (5).

Globally, 10% of pregnant women experience antenatal depression, which extended to 15.6% in developing countries (5). The magnitude of antenatal depression varies within sub-Saharan African countries, which ranges from 9.9% in Ghana to 47% in rural South Africa (5). Pregnant women living in low-and middle-income countries are known to be at high risk of antenatal depression (1). A study suggested that perinatal women from rural areas are at higher risk of depression and anxiety than their urban peers (6).

In Ethiopia, approximately one-quarter of pregnant women face depression during pregnancy (5). Studies show that depressive symptoms are more frequent during pregnancy than during the postpartum period, and depressive symptoms during pregnancy may have devastating consequences, not only for the women but also for the child and family (7).

Antenatal depression has been linked to negative health-related behaviors and outcomes, including poor nutrition, increased substance use, inadequate prenatal care, miscarriages, pre-eclampsia, preterm delivery, low birth weight, impairments in mother–infant interactions, postnatal depression, and suicide (8). Loss of motivation and interest in common activities including self-care; reduced social support, reduced adherence to healthcare practitioner recommendations, and disability that are linked to depression might negatively impact the healthcare service use of the mothers (9).

Evidence suggested that having low social support, intimate partner violence, anxiety during pregnancy, stressful life events, low self-esteem, and a previous history of depression were the psychological risk factors influencing antenatal depression (5, 10, 11). Unplanned pregnancy, the experience of abortion, complications at an earlier delivery, and a history of cesarean delivery were the obstetric-related factors that increased the risk of depression during pregnancy (10). In addition to this being single, marital dissatisfaction, low family income, and absence of paid work increase the likelihood of having antenatal depression (5, 11, 12).

Integrating early screening, detection, and treatment of antenatal depression into routine antenatal care is the key strategy to reduce the burden of morbidity and disability associated with antenatal depression (2, 7, 13).

Nonetheless, maternal mental healthcare is not well-integrated, and healthcare providers emphasize less on antenatal depression. In addition, most of the studies conducted are institutional study designs, which may not show the real prevalence and risk factors, because evidence shows that 29% of rural pregnant women and 15% of urban pregnant women had no ANC visit (14).

To better understand the problem of antenatal depression, more research is needed to alleviate the problem and the factors associated with it. Even though it is necessary to identify and understand any inequalities between rural and urban areas of antenatal depression, per our literature review, there was a lack of information research in this area. To the best of the researcher’s knowledge, no study has specifically addressed the association between the rural–urban status and antenatal depression in this study area, and few of the studies previously conducted in another were based on exclusively rural or urban samples, which hinders comparison between the two groups and associated factors.

Therefore, the study aimed to assess the association between living in a rural or urban area and the risk for antenatal depression in women from Gimbi, West Wollega Zone, Ethiopia.

## 2 Methods

### 2.1 Study area, period, and design

A community-based comparative cross-sectional study design was conducted from 10 February 2023 to 15 March 2023. The study was conducted in Gimbi district, western Ethiopia, 441 km from Addis Ababa, Ethiopia. The study area has 31 rural and 6 urban kebeles, and according to population projection for 2022, there were 166,054 (82,861 male individuals and 83,193 female individuals) residents (15). In the district, there are different ethnic groups and different governmental and non-governmental health institutions. One governmental hospital, an NGO hospital, 1 Family Guidance of Ethiopian Association, 12 medium clinics, and 5 health centers in the district provide healthcare services including antenatal care for the population living in this study area (15).

### 2.2 Participants

All pregnant women living in Gimbi district and all pregnant women in selected kebeles of Gimbi district during the study period were the source population and study population of the study, respectively. All pregnant women of any gestational age living in the Gimbi district during the study period were included in the study. Pregnant women who were critically ill and unable to communicate during the time of data collection were excluded from the study.

### 2.3 Sample size determination

The sample size was determined using a double population proportion formula by considering the following assumptions: level of significance (0.05), power (0.80), the expected proportion of pregnant mothers tested positive for depression in urban (16.6%) (16), and the expected proportion of pregnant mothers tested positive for depression in rural (28.7%) (9). A non-response rate of 10% and a design effect of 2 were considered.

$$\begin{array}{l} n={{\left(Z_{/2}+Z_{\beta }\right)}^{2}}_{*}\;\left(p_{1}\;{\left(1-p_{1}\right)}_{+}\;p_{2}\;\left(1-p_{2}\right)\right)/{\left(p_{1}-p_{2}\right)}^{2};\\ n={{\left(1.96+0.84\right)}^{2}}_{*}\;(0.287{\left(1–0.287\right)}_{+}\;0.166\left(1–0.166\right)/\\ {\left(0.287–0.166\right)}^{2}=124\;\mathrm{for urban population and}\;124\;\\ \mathrm{for rural population}\text{.} \end{array}$$

where, Zα = the z-score corresponding to the 95% confidence level, which is 1.96.

Z_*β* =_ is the critical value of the normal distribution at β, which is 0.84.

Epi Info version 7.2.5.0 was used to calculate the sample size for the factor variable, using the following assumptions: margin of error (0.05), power of study (0.80), percent among exposed^1^ (65.7%), and percent among non-exposed (39.8%), and ratio of 1 for social support as associated factors for antenatal depression (16). The Epi Info sample size calculator produced 130 for urban and 130 for rural. Since the multistage sampling technique was used, the sample size was multiplied by the design effect of 2. Therefore, the final sample size was calculated to be 520, 260 for urban participants and 260 for rural participants.

### 2.4 Sampling procedure

A multistage sampling method was used. Gimbi district has 37 kebeles; out of these, 31 are rural kebeles and 6 are urban kebeles. Three kebeles were selected by simple random sampling from 31 rural kebeles, 10 kebeles, and 6 urban kebeles. The households with pregnant women were taken from CHIS (Community Health Information System) in rural kebeles and from family health registration for urban kebeles. A systematic random sampling method was used to select the household with pregnant women. The first household was selected using the lottery method. The next samples were taken in the interval of three households in rural areas and every four households in urban areas until the required numbers of eligible pregnant women were recruited in the study area.

In cases where the respondents were not available at home at the time of data collection, repeat visits were made at least three times, and for the household with more than one pregnant woman, only one was sampled randomly.

### 2.5 The study variables

Dependent variable was antenatal depression among pregnant women.

#### 2.5.1 Independent variables

Socio-demographic characteristics of pregnant women were age, marital status, occupational status of the mother and partner, educational status of the mother, educational status of the husband/partner, ethnicity, and residence.

Obstetric factors were the number of live babies, history of abortion, need of pregnancy, complication during pregnancy, type of complications, ANC follow-up, the number of ANC visits, social support, intimate partner violence, substance use, and previous history of depression.

### 2.6 Operational definitions

Antenatal depression is a form of clinical depression that is measured using the Edinburgh Postnatal Depression Scale (EPDS). EPDS was validated in Addis Ababa for antenatal and postpartum use and showed a sensitivity of 84.6% and a specificity of 77.0% (17). Accordingly, women with EPDS of ≥13 were assigned to have ‘antenatal depression’ (i.e., labeled as ‘Yes’). Those women with EPDS of <13 were reported to have ‘no antenatal depression’ (i.e., labeled as ‘No’) (17, 18).

**Maternity Social Support Scale (MSSS)** is a six-item, self-report measure of maternal perceptions of social support, care, and love from their family. The tool is a 5-point Likert scale with a maximum score of 30. Each item was rated as 5 for always, 4 for most of the time, 3 for some of the time, 2 for rarely, and 1 for never. Accordingly, social support was classified into three categories: high social support (scores 24–30), medium social support (scores 18–23), and low social support (below 18) categories (16, 19).

**Intimate partner violence (IPV)** is abuse that occurs in a romantic relationship. It refers to both current and former spouses and dating partners. IPV was considered if a pregnant woman reported one of the following during the current pregnancy: husband/partner done things to scare or intimidate on purpose or threatened to hurt someone they care about or hit, slapped, or thrown something that could hurt her or forced or pressured to have sexual intercourse when she did not want to (20, 21).

Complications during pregnancy: Problems during pregnancy include physical and mental conditions that affect the health of the mother or the baby. It can be caused by or worse by being pregnant (22).

### 2.7 Data collection tool and procedure

Data were collected from study participants using the interviewer-administered structured and pretested questionnaire. The questionnaire contains socio-demographic factors, obstetric history, social support factors, substance use, intimate partner violence, previous history of depression, and depressive symptoms, which were assessed using the Edinburgh Postnatal Depression Scale (EPDS). EPDS is a 10-item questionnaire, scored from 0 up to 3. A higher EPDS score indicates more depressive symptoms and the tool has been validated for detecting depression in antepartum and postpartum samples in many countries. The instrument was validated in Addis Ababa for antenatal and postpartum use and showed a sensitivity of 84.6% and specificity of 77.0% (20). The Maternity Social Support Scale (MSSS) was used to measure the respondent’s social support status. The data collection tool was prepared in English and translated into the Afan Oromo. To check the consistency of the idea, another individual again translated the tool back into English.

For the data collection process, five diploma midwives data collectors and one BSc psychiatric nursing supervisor were trained in data collection and supervision of the data collection. Data collectors and supervisors received 1-day training on the purpose of the study and data collection techniques.

A pretest was conducted in Lalo Asabi District in Inango 01 and 02 Kebele and Haroji Serdo and Dongoro Keta among 5% (24 study participants) of the sample size to check the respondent’s response, the language’s clarity, and the tool’s appropriateness. At the end of the pretest, necessary correction measures and amendments were made. Data were checked for accuracy, completeness, consistencies, and missed values and variables each day after the field.

### 2.8 Data analysis technique

The collected data were coded and entered into Epi data before the analysis. Then, the data were exported to SPSS and analyzed using version 24. Descriptive statistics were performed, and the corresponding results were described in frequencies and percentages and then reported in text, graphs, and tables.

The mean and standard deviation were calculated for continuous variables. A binary logistic regression analysis was used to identify factors that affect antenatal depression. Variables with a *p*-value of 0.25 or less in the bi-variable logistic regression model were candidates for the multivariable logistic regression model to control the possible confounders. Multicollinearity was checked using a variance inflation factor (VIF) for all independent variables. The strength of the association of predictor variables was assessed using OR, and the significance of variables was reported using 95% CI and *p*-values less than 0.05. Model fitness was assessed using the Hosmer–Lemeshow goodness-of-fit test (accepted with *p* > 0.05). Histogram and skewness were used for the normality check.

## 3 Results

### 3.1 Socio-demographic characteristics of study participants

A total of 520 participants, 260 urban and 260 rural respondents, participated in the interview, making the response rate 100%. The mean age of the respondents was 25.59 (±5SD) and 26 (±4.9SD) years for urban and rural participants, respectively. Overall, 243 (93.5%) urban and 256 (98.5%) rural study participants were married. Among respondents, 235 (90.4%) and 240 (92.3%) were Oromo, while 25 (9.6%) and 20 (7.7%) were from other ethnic groups for urban and rural, respectively (Table 1).

**Table 1:** Socio-demographic characteristics of the study participants among urban and rural pregnant women in Gimbi District, West Wallaga, Ethiopia, 2023 (*N* = 260 urban and 260 Rural).

Categories		Urban rural		
		Frequency (%)	Frequency (%)	*p*-value
Religion	Protestant	149 (57.31%)	123 (47.31%)	
	Orthodox	56 (21.54%)	106 (40, 77%)	0.001
	Muslim	38 (14.62%)	29 (11.15%)	
	Others	17 (6.53%)	2 (0.77%)	
Educational status (Mothers)	Unable to read and write	10 (3.85%)	41 (15.77%)	
	Read and write	17 (6.54%)	44 (16.92%)	0.001
	Primary school (1–8)	66 (25.38%)	90 (34.62%)	
	Secondary school (9–12)	94 (36.15%)	71 (27.31%)	
	College and above	73 (28.08%)	14 (5.38%)	
Educational status (Husband)	Unable to read and write	9 (3.46%)	26 (10%)	
	Read and write	10 (3.85%)	29 (11.15%)	
	Primary school (1–8)	41 (15.77%)	51 (19.62%)	
	Secondary school (9–12)	110 (42.31%)	116 (44.62%)	0.001
	College and above	90 (34.62%)	38 (14.62%)	
Current Occupation (Mother)	Government employee	49 (18.85%)	10 (3.85%)	
	Merchant	41 (15.77%)	40 (15.38%)	
	Student	43 (16.54%)	30 (11.54%)	0.001
	Housewife	111 (42.69%)	148 (56.92%)	
	Others	16 (6.15%)	32 (12.31%)	
Current Occupation (Husbands)	Government employee	84 (32.31%)	16 (6.15%)	
	Farmer	12 (4.62%)	83 (31.92%)	0.001
	Merchant	54 (20.77%)	95 (36.54%)	
	Self-employed	92 (35.38%)	63 (24.23%)	
	Others	18 (6.92%)	3 (1.15%)	

Others = private workers, daily laborers, no known occupation.

### 3.2 Obstetric characteristics of the study participants

This study found that 174 (66.92%) rural and 195 (75%) urban participants had been pregnant before. Approximately 82 (31.54%) urban and 89 (34.23%) rural participants were gravida one. Among urban participants, 66 (25.88%) and 41 (15.77%) of rural participants had complications during the index pregnancy. Of the respondents, 222 (85.38%) urban and 246 (94.62%) rural respondents planned their pregnancy, while 114 (43.85%) urban and 39 (15%) rural respondents expressed fear giving birth to the current pregnancy (Table 2).

**Table 2:** Obstetric characteristics of the study participants among urban and rural pregnant women in Gimbi District, West Wallaga, Ethiopia, 2023 (*N* = 260 urban and 260 Rural).

		Urban		Rural		p-value
		Number	Percentage	Number	Percentage	
History of abortion	Yes	23	8.85	60	23.08	0.01
	No	237	91.15	200	76.92	
Number of abortions	<=1	20	86.9	54	90	0.67
	> = 2	3	13.04	6	10	
Complications during the index pregnancy	Yes	66	25.38	41	15.77	0.001
	No	194	74.62	219	84.23	
vaginal bleeding	Yes	22	8.46	24	9.23	0.001
	No	238	91.54	236	90.77	
Blurring of version	Yes	13	5.00	12	4.62	0.001
	No	247	95.00	248	95.38	
Swollen hand and face	Yes	22	8.46	2	0.77	0.001
	No	238	91.54	258	99.23	
Planned pregnancy	Yes	222	85.38	246	94.62	0.001
	No	38	14.62	14	5.38	
ANC follow up	Yes	245	94.23	229	80.08	0.013
	No	15	5.77	31	11.92	
Number of ANC visits	<=3	180	69	195	75	0.001
	> = 4	65	25	34	13.1	
	No ANC	15	5.7	31	11.9	
Fear of giving birth to the current pregnancy	Yes	114	43.85	39	15.00	0.001
	No	146	56.15	221	85.00	
What is the desired sex of your current fetus?	Male	78	30.00	31	11.92	0.001
	Female	79	30.38	28	10.77	
	Not identified	103	39.62	201	77.31	

### 3.3 Maternity social support and intimate partner violence among study participants of urban and rural pregnant women

Approximately 165 (63.5%) rural and 124 (47.7%) urban study participants reported that they had medium maternity social support during their current pregnancy, while 5 (1.9%) and 12 (4.6%) reported that they had high maternity social support. In total, 19 (34.6%) rural and 124 (23.8%) urban participants reported that they have low social support.

**Figure 1:**
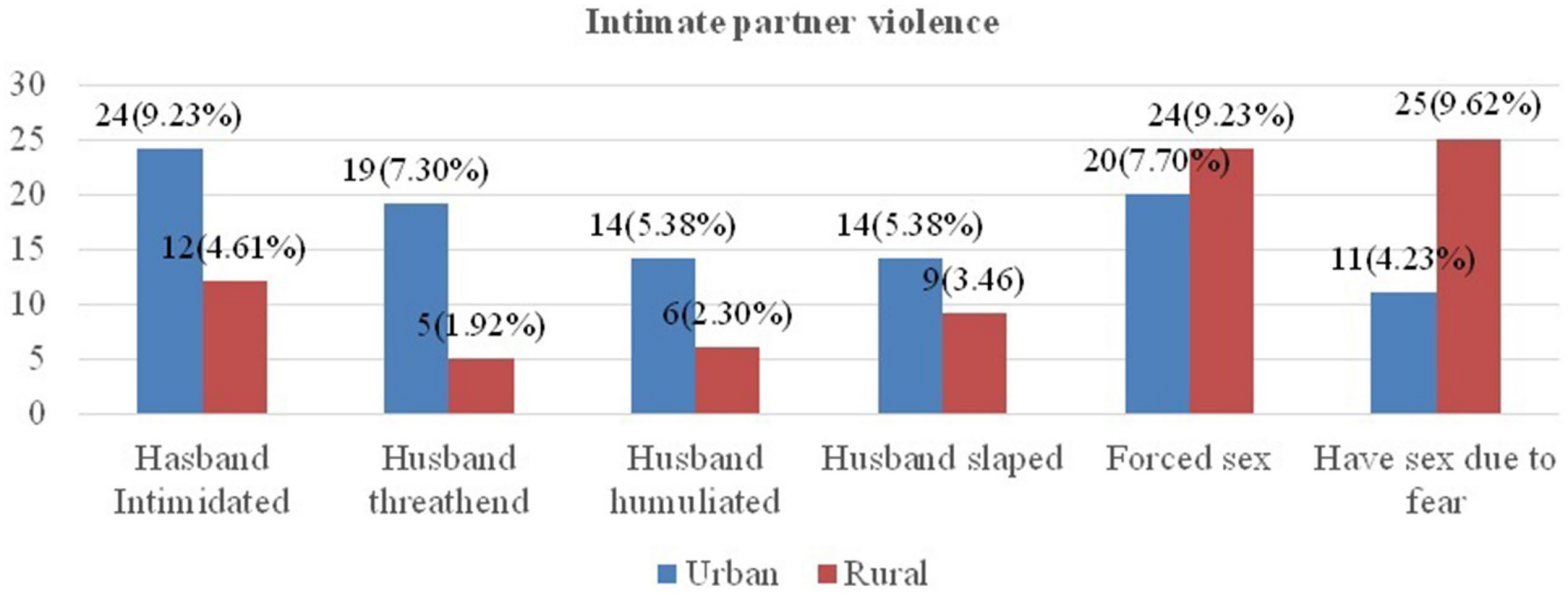
Intimate partner violence among urban and rural pregnant women in Gimbi District, West Wallaga, Ethiopia, 2023.

In this study area, 23 (8.85%) urban and 10 (3.85%) rural participants reported that their husbands/partners insulted them during their current pregnancy. Among respondents, 19 (7.3%) urban and 5 (1.92%) rural study participants reported that their husbands threatened them during their current pregnancy, and 14 (5.38%) and 9 (3.46%) study participants had been slapped by their husbands in urban and rural areas, respectively (Figure 1).

### 3.4 History of depression, substance use, and antenatal depression status of the study participants among urban and rural pregnant women

This study revealed that 25 (9.62%) and 6 (2.31%) of urban and rural respondents mentioned that they had a history of depression in their lives, respectively. Regarding the family history of depression, 14 (5.4%) and 13 (5%) urban and rural respondents’ families or relatives had previous history of depression, respectively. The finding of this study showed that the prevalence of antenatal depression was 56 (21.5%), [95% CI 16.9–25.5] among urban and 19.2% 50 (95%) (CI 14.6–23.8) rural areas.

Regarding substance use, this study showed 8 (3%) urban and 5 (1.9%) rural study participants reported that they chewed chat during the current pregnancy. Approximately 12 (4.6%) and 6 (2.3%) pregnant mothers drink alcohol in urban and rural, respectively. Overall, 51 (19.6%) urban and 36 (13.6%) rural husbands of pregnant mothers chew chat.

### 3.5 Factors associated with antenatal depression among urban pregnant women

Chi-square and binary logistic regression analyses were performed to identify the candidate variables for multivariable logistic regression. Accordingly, variables with a *p*-value <0.25 in bi-variable logistic regression were level of education, husband consuming alcohol, having complications during pregnancy, sex of the current fetus, husband hurts during pregnancy, and ever had depression.

### 3.6 Multivariable binary logistic regression analyses for urban pregnant women

Variables with a *p*-value <0.25 in bi-variable logistic regression were transferred to multivariable logistic regression to remove confounding variables. Multicollinearity was checked using a variance inflation factor (VIF) for all independent variables, the maximum value of VIF was 1.8, and none of the explanatory variables correlated. Hosmer–Lemeshow goodness-of-fit test was used to test for model fitness and the model was fit at 0.55 *p*-values. Accordingly, husbands who drank alcohol were 3.78 times (AOR: 3.78, 95% CI: 1.24, 11.49) more likely to develop antenatal depression (AND) when compared to their counterparts. Similarly, pregnant women who had complications during index pregnancy were 4.92 times (AOR: 4.92, 95% CI: 1.35, 17.88) more likely to experience antenatal depression when compared to their counterparts.

The odds of antenatal depression among pregnant women who can read and write were 2.14 times more likely [AOR: 2.14, 95% CI: (1.05, 4.67)] to develop AND when compared to the pregnant women whose educational level was college and above. Furthermore, pregnant women who ever had depression in their life were 3.20 times more likely [AOR: 3.20, 95% CI: 1.30, 7.85] to develop AND when compared to their counterparts among study participants in urban areas (Table 3).

**Table 3:** Multivariable logistic regression analysis of antenatal depression and associated factors among urban, Gimbi district, 2023.

Variables		Antenatal Depression				
		Yes (%)	No (%)	COR (95% CI)	AOR (95% CI)	*P*-value
Husband drink alcohol	Yes	22 (31)	49 (69)	2.05 (1.01, 3.82)	3.78 (1.24, 11.49)	0.02^*^
	No	34 (18)	155 (82)	1		
Level of education	Cannot read and write	2 (20)	8 (80)	0.97 (0.18, 5.03)	2.8 (0.16, 4.81)	0.48
	Can read and write	5 (29.41)	12 (70.59)	1.6 (0.49, 5.28)	2.14 (1.05, 4.67)	0.04^*^
	Primary	21 (31.8)	45 (68.18)	1.8 (0.83, 3.8)	3.61 (0.53, 2.42)	0.18
	Secondary	13 (13.82)	81 (86.18)	0.62 (0.27, 1.40)	1.60 (0.24, 4.64)	0.62
	College and above	15 (20.55)	58 (79.45)	1		
Complication during pregnancy	Yes	19 (28.79)	47 (71.1)	1.75 (0.9, 3.2)	4.92 (1.35, 17.88)	0.015^*^
	No	37 (19.07)	157 (80.93)	1		
Sex of current fetus	Male	21 (26.92)	57 (73.08)	2.34 (1.10, 4.97)	0.53 (0.09, 2.8)	0.45
	Female	21 (26.6)	58 (73.4)	2.30 (1.08, 4.88)	0.45 (0.09, 2.01)	0.31
	Not Identified	14 (13.6)	89 (86.4)	1		
Planned pregnancy	Yes	43 (19.37)	179 (80.63)	0.46 (0.22, 0.98)	0.25 (0.21, 1.05)	0.06
	No	13 (34.21)	25 (65.79)	1		
Husband hurt during pregnancy	Yes	8 (42.1)	11 (57.9)	2.92 (1.11, 7.66)	2.35 (0.81, 6.82)	0.11
	No	48 (19.91)	193 (80.09)	1		
Ever had depression	Yes	11 (44)	14 (56)	3.32 (1.41, 7.79)	3.20 (1.30, 7.85)	0.01*
	No	45 (19.15)	190 (80.85)	1		

1-reference.

### 3.7 Factors associated with antenatal depression among rural pregnant women

Chi-square and binary logistic regression analyses were used to identify the candidate variables for multivariable logistic regression. Variables with a *p*-value <0.25 in bi-variable logistic regression were husband consuming alcohol, complication during pregnancy, sex of current fetus, husband hurts during pregnancy, ANC follow-up, forced sex, planned pregnancy, and ever had depression.

### 3.8 Multivariable binary logistic regression analyses among rural pregnant women

Variables with a *p*-value <0.25 in bi-variable logistic regression were transferred to multivariable logistic regression to remove confounding variables. Multicollinearity was checked using a variance inflation factor (VIF) for all independent variables, the maximum value of VIF was 1.55, and none of the explanatory variables correlated. Hosmer–Lemeshow goodness-of-fit test was used to test for model fitness and the model was fit at 0.65 *p*-values.

Having complications during pregnancy, having a history of depression, ANC follow-up, and planned pregnancy were significantly associated with AND among rural women. Accordingly, the odds of AND among pregnant women who had complications during pregnancy were 2.77 times (AOR: 2.77, 95% CI: 1.30, 5.92) more likely to develop AND when compared to their counterparts. Similarly, pregnant women who had ever had depression in their lives were 2.84 times more likely (AOR: 2.84, 95% CI: 1.15, 7.04) to develop AND when compared to their counterparts. The odds of AND among pregnant women who had no planned pregnancy were 4.51 times more likely (AOR: 4.51, 95% CI: 1.10, 1.86) to develop AND when compared to women who had a plan, and the odds of AND among pregnant women who had no ANC follow-up were 6.6 times (AOR: 6.6, 95% CI: 2.63, 16.85) more likely to develop AND when compared to pregnant women who ever had ANC follow-up (Table 4).

**Table 4:** Multivariable logistic regression analysis of antenatal depression and associated factors among rural Gimbi district, 2023.

Variables	Category	Antenatal depression				
		Yes (%)	No (%)	COR (95% CI)	AOR (95% CI)	*P*-value
Complication during pregnancy	Yes	17 (26.56)	47 (73.44)	2.08 (1.05, 4.11)	**2.77 (1.30, 5.92)**	0.008^**^
	No	29 (14.80)	167 (85.20)	1		
Sex of current fetus	Male	19 (24.35)	59 (75.65)	2.22 (1.02, 4.85)	1.47 (0.36, 5.90)	0.58
	Female	18 (27.78)	61 (77.22)	2.04 (0.93, 4.47)	0.99 (0.21, 4.49)	0.98
	Not identified	13 (12.62)	90 (87.38)	1		
ANC follow up	Yes	32 (13.97)	197 (86.03)	1		
	No	14 (45.16)	17 (54.84)	5 (2.28, 11.28)	**6.6 (2.63, 16.85)**	0.001^***^
Ever had depression	Yes	10 (40)	15 (60)	3.25 (1.36, 7.75)	2.84 (1.15, 7.04)	0.024^*^
	No	40 (17.02)	195 (82.98)	1		
Forced sex	Yes	6 (30)	14 (70)	1.91 (0.7, 5.24)	2.09 (0.53, 8.16)	0.28
	No	44 (18.33)	196 (81.77)	1		
Planned pregnancy	Yes	40 (18.02)	182 (89.98)	1		
	No	10 (26.32)	28 (73.68)	1.63 (0.73, 3.61)	**4.51 (1.10, 1.86)**	0.03^*^
Husband hurt during pregnancy	Yes	7 (36.84)	12 (63.16)	2.6 (0.9, 7.2)	2.06 (0.49, 3.53)	0.32
	No	43 (17.84)	198 (82.16)	1		
Husband drink alcohol	Yes	19 (26.76)	52 (73.24)	1.86 (0.97, 3.57)	2.00 (0.47, 8.53)	0.34
	No	31(16.40)	158(83.60)	1		

1-reference ***p-value < 0.0001, **p-value < 0.05. Bolded values describe the statistically significant AOR.

## 4 Discussion

This study revealed that the prevalence of antenatal depression was 56 (21.5%), [95% CI 16.9–25.5] and 50 (19.2%) [95% CI 14.6–23.8] among urban and rural participants, respectively. Having complications during pregnancy, previous history of depression, having a spouse consuming alcohol, and low educational status were found to be factors influencing antenatal depression among urban pregnant women. No antenatal care follow-up, unplanned pregnancy, and during pregnancy were identified as factors associated with antenatal depression among rural pregnant women in this study area.

The prevalence of antenatal depression among urban mothers was higher than the prevalence of antenatal depression among rural mothers in the Gimbi district. This finding is similar to the study conducted in Goba town and Sodo rural district, where the urban area is higher than rural areas (4, 9). However, the comparative study conducted in the UK revealed that rural prevalence is higher than urban counterparts (6). The findings could be due to methodological, social, and economic differences. In addition, having a spouse who consumes alcohol is a factor among urban pregnant women in this study area, which might have an impact to increase the possibility of facing depression. Because the spouses consuming alcohol spend money on alcohol consumption, the pregnant woman is bound to suffer from depression when she loses her expenses for herself and the family, as well as for the childcare.

In this study, the prevalence of antenatal depression among urban pregnant women was higher than in Northwest Ethiopia at 15.20% (5), Debra Tabor town at 11.8% (13), Jimma town at 16.6% (16), Northeast Ethiopia at 17.9% (23), Tanzania at 15% (8), and Low and Middle Income Countries (LMIC) at 16% (24). However, this study finding showed a lower prevalence than the study conducted in Addis Ababa, which was 24.94% (7), 24.3% in Somali (25), 23% in Gondar (26), 31.5% in Southeast Ethiopia (27), 24.5% in Vietnam (25), 35.7% in Bangalore (26), and 24.5% in Nigeria (8). The possible reason for the difference in findings could be the difference in socio-economic status and study approach where the majority of these studies have been institution-based, and the current study is a community-based approach.

This study revealed that the prevalence of antenatal depression among rural participants was 19.2%, which was less than that found in studies conducted in rural South Africa (47%) (27), in rural Sodo district, southern Ethiopia, (28.7%) (9), and in West Shoa (32.3%) (2). The difference could be because the study conducted in South Africa was among the population with a high prevalence of HIV, which could put pregnant mothers at high risk of developing antenatal depression. On the other hand, this study revealed that antenatal depression in this study area was less than in southern Ethiopia. This could be because the study conducted in southern Ethiopia was among pregnant women in the second and third trimesters and followed them for weeks, which could capture more cases because antenatal depression is common in the second and third trimesters of the pregnancy (28). The current study included all pregnant women regardless of gestational age. However, the finding of this study is greater than in rural China 13% (29); this could be due to the difference in the quality of maternal health service, which is by far better in China.

This study revealed that pregnant mothers whose spouses consume alcohol were positively associated with antenatal depression in urban. This finding was in line with the study conducted in Northwest Ethiopia (5). This might be where the husbands spend money on alcohol consumption, the family has nothing to use, and, in this regard, the pregnant woman is bound to suffer from depression when she loses her expenses for herself and the family, as well as for the child care.

This study also revealed that pregnant women who can read and write have higher odds of antenatal depression when compared to those who have attended college and above among urban respondents. This finding is consistent with the findings of the study conducted in the Somali region of Ethiopia and China, where a lower educational level is the risk factor for antenatal depression (30, 31). In contrast to this, other findings from a study conducted in Awabale Woreda, Northwest Ethiopia (32), and a study entitled “Educational differences in prenatal anxiety and depressive symptoms and the role of childhood circumstances” (33) revealed that higher education level is associated with a higher risk of maternal depression.

According to this study, complications during pregnancy were positively associated with antenatal depression. This is in line with the study of Debre Tabor Town (34). This may be because pregnant women who have suffered complications might think of the complications, which could worsen their mental health status.

A history of depression was found to be highly associated with antenatal depression among pregnant women. Mothers who had a previous history of depression were more likely to develop antenatal depression among urban pregnant women. This study finding is similar to the study conducted in Southwest Ethiopia, where the previous history of depression is significantly associated with antenatal depression (4). The possible explanation for this finding could be that pregnant women who have experienced depression in the past, especially during pregnancy, are more likely to develop depression again because they worry about their past illness.

Having unplanned pregnancies was also another factor associated with antenatal depression among rural participants. This finding is supported by the study conducted in Southwest Ethiopia, where unplanned pregnancy was positively associated with antenatal depression (4). The reason for this finding would be that unplanned pregnancy can cause health, social, and psychological problems. When there is an unplanned pregnancy, the pregnant woman will be out of education and development work, and she waits only for her husband’s hand and will not be able to support herself as well as her family. This may cause depression during pregnancy. In addition, women encountering unwanted pregnancy could have unfavorable attitudes toward seeking healthcare services and be prone to face depression (35).

Furthermore, this study also revealed that no ANC follow-up was significantly associated with antenatal depression among rural respondents. This finding is similar to the study conducted in Northwest Ethiopia (34). According to the current study finding, the chance of developing antenatal depression is high among pregnant women who have no ANC follow-up. This may be because pregnant women who did not receive ANC follow-up during pregnancy may worry much and the risk of antennal depression is even higher because the health status of the unborn infant and the mother is unknown. ANC follow-up could be the opportunity for pregnant women to get counseling, early detection, and treatment of any mental illness as early as possible.

This study aimed to compare the prevalence of antenatal depression among urban and rural pregnant mothers. The study used a community-based cross-sectional study design, where having the community as the study setting increases the chance of capturing both urban and rural women attending antenatal care or not attending antenatal care visits. However, this study may not declare a temporal relationship between cause and effect due to the nature of the cross-sectional study design—the possibility of social desirability bias to some sensitive variables such as substance use and partner violence. The study considered the last 7 days as a reference period to assess antenatal depression, which might have introduced recall bias. In addition, a systematic random sampling method was used to select the study participants despite the availability of the sampling frame. However, the effect of some of the limitations has been minimized by different approaches. For example, to reduce the limitation related to sensitive variables, the data collectors were trained to interview the respondents in quiet and private places. To minimize the recall biases, the respondents have been interviewed by mentioning important and unforgotten days such as holidays.

## 5 Conclusion

The prevalence of antenatal depression in urban participants was higher than the prevalence of antenatal depression in rural participants in the Gimbi district. Spousal consuming alcohol behavior, complications during index pregnancy, level of education (can read and write), and having a previous history of depression were the factors significantly associated with antenatal depression among urban respondents. No ANC follow-up, complications during index pregnancy, and unplanned pregnancies were significantly associated with antenatal depression among rural respondents. Therefore, health workers need to know about antenatal depression’s magnitude and risk factors and integrate them as part of their overall assessment. Integrating early screening, detection, and treatment of antenatal depression into routine antenatal care can improve the morbidity related to the problem. The district health office should monitor the antenatal service delivery and ensure that all women in the district have access to antenatal services. The district family health department should provide contraceptive methods for women of reproductive age groups to reduce the risk of unplanned pregnancy in the district, particularly for women living in the rural area of the district. The regional, zonal, and district health authorities should initiate information education and communication platforms addressing antenatal depression. In addition, the government and the local authority should work more to ensure universal education in the district.

## Data Availability

The raw data supporting the conclusions of this article will be made available by the authors, without undue reservation.

## Footnotes

^1^Exposed is the group exposed to the outcome of interest (Antenatal depression) whereas non-exposed is the group free from the outcome of interest (Antenatal depression).
